# Progenitors Mobilized by Gamma-Tocotrienol as an Effective Radiation Countermeasure

**DOI:** 10.1371/journal.pone.0114078

**Published:** 2014-11-25

**Authors:** Vijay K. Singh, Stephen Y. Wise, Oluseyi O. Fatanmi, Jessica Scott, Patricia L. P. Romaine, Victoria L. Newman, Amit Verma, Thomas B. Elliott, Thomas M. Seed

**Affiliations:** 1 Armed Forces Radiobiology Research Institute, Bethesda, Maryland, United States of America; 2 Department of Radiation Biology, F. Edward Hébert School of Medicine, Uniformed Services University of the Health Sciences, Bethesda, Maryland, United States of America; 3 Tech Micro Services, Bethesda, Maryland, United States of America; French Blood Institute, France

## Abstract

The purpose of this study was to elucidate the role of gamma-tocotrienol (GT3)-mobilized progenitors in mitigating damage to mice exposed to a supralethal dose of cobalt-60 gamma-radiation. CD2F1 mice were transfused 24 h post-irradiation with whole blood or isolated peripheral blood mononuclear cells (PBMC) from donors that had received GT3 72 h prior to blood collection and recipient mice were monitored for 30 days. To understand the role of GT3-induced granulocyte colony-stimulating factor (G-CSF) in mobilizing progenitors, donor mice were administered a neutralizing antibody specific to G-CSF or its isotype before blood collection. Bacterial translocation from gut to heart, spleen and liver of irradiated recipient mice was evaluated by bacterial culture on enriched and selective agar media. Endotoxin in serum samples also was measured. We also analyzed the colony-forming units in the spleens of irradiated mice. Our results demonstrate that whole blood or PBMC from GT3-administered mice mitigated radiation injury when administered 24 h post-irradiation. Furthermore, administration of a G-CSF antibody to GT3-injected mice abrogated the efficacy of blood or PBMC obtained from such donors. Additionally, GT3-mobilized PBMC inhibited the translocation of intestinal bacteria to the heart, spleen, and liver, and increased colony forming unit-spleen (CFU-S) numbers in irradiated mice. Our data suggests that GT3 induces G-CSF, which mobilizes progenitors and these progenitors mitigate radiation injury in recipient mice. This approach using mobilized progenitor cells from GT3-injected donors could be a potential treatment for humans exposed to high doses of radiation.

## Introduction

Radiological and nuclear mass-casualty events are significant threats to civilian populations and deployed members of the military. Disasters that occurred at the Chernobyl Nuclear Power Plant in 1986 and more recently at the Fukushima Daiichi Nuclear Power Plant in 2011 highlight the need for effective treatments to contend with the damaging effects of ionizing radiation [1]–[4]. In addition to accidents that might occur at sites utilizing radioactive materials, the Nation must prepare for the inevitability of a terrorist group obtaining radioactive material from the more than 100 countries that do not have adequate regulatory control or monitoring systems and detonating a ‘dirty bomb’ [5]. In the worst possible scenario, such a bomb or an improvised nuclear device would be detonated in an urban setting, inciting not only fear and panic, but an array of medical problems and deaths resulting from the initial blast, intense heat and subsequent radioactive fallout. Those affected would be in need of immediate medical treatment [6], [7].

Significant acute radiation injury in humans occurs at whole-body doses above 1 Gy, with symptoms getting progressively more severe as the level of radiation exposure increases [8]. A dose range of 1 to 8 Gy is characterized by the loss of hematopoietic cell regenerative ability resulting in the “hematopoietic syndrome.” The numbers of red and white blood cells, neutrophils, platelets as well as others decline and susceptibility to potentially fatal infections increase. In the exposure range of ∼8 to 30 Gy, hematopoietic symptoms are present in addition to symptoms caused by significant breakdown of the gastrointestinal (GI) system which results in the “GI Syndrome.” Breakdown of the GI system results in translocation of GI bacteria to other organs, which ultimately results in sepsis and eventually death. Collectively, hematopoietic and GI syndromes are well recognized as the major subsyndromes of the acute radiation syndrome (ARS). At doses significantly above 8 Gy, significant damage is done to the nervous system that unconditionally results in rapid death [8]. Because damage resulting from such extremely high radiation exposure has been deemed untreatable, the scientific community has focused its efforts on finding preventative and mitigating treatments for ARS. The search for treatments to counter potentially lethal radiation injury has been underway for the past several decades, resulting in multiple classes of radiation countermeasures [9]–[12]. However, to date there have been no suitable countermeasures approved for use by the U.S. Food and Drug administration (US FDA) for the treatment of ARS.

Most recently, natural products have been investigated for prevention and therapy of human diseases because they are ‘generally recognized as safe’ and appropriate for medicinal purposes. Unlike their synthetic analogs, they are well tolerated and minimally toxic, even in the upper ranges of dietary intake [13], [14]. Such a vitamin that has been introduced to the research spotlight is vitamin E, which is well known for its antioxidant, neuroprotective, and anti-inflammatory properties [14]. Vitamin E is a family of eight compounds that are collectively known as tocols. Tocols exist as four tocopherols (α, β, γ, δ) and four tocotrienols (α, β, γ, δ) [15]. The isomeric forms of both groups are differentiated by the position of the methyl groups on the chromanol ring. All tocols have powerful antioxidant activity that helps regulate peroxidation reactions and control free radical production within the body [16], [17]. These compounds also help protect cells from increased oxidative damage caused by free radicals produced by radiation exposure.

Although the majority of investigations have used tocopherols (more specifically α-tocopherol) in tests of radioprotection, recent discoveries indicate that the therapeutic targets are distinct between the two groups, signifying that the members of the vitamin E family largely work through different mechanisms and do not impart biological functions that significantly overlap and are redundant [18], [19]. Tocotrienols have clearly distinct functions in maintaining health and treating disease [20]. A number of studies have shown that tocotrienols are superior antioxidants compare to tocopherols [21]–[24]. 

Recently, it has been demonstrated that tocopherol succinate, δ-tocotrienol, and γ-tocotrienol (GT3) protect mice against radiation injury[9], [25]–[27] and the radioprotective efficacy of tocols is mediated largely through granulocyte colony-stimulating factor (G-CSF) [28]–[31]. G-CSF is known to mobilize progenitors into circulation and there is a recent report that GT3 induces G-CSF and mobilizes progenitors as well [29], [32], which confirms an earlier suggestion that tocols in general have a bone marrow progenitor-mobilizing capacity [33]. With these results in mind and because we now recognize GT3's radioprotective superiority, in this study we sought to extend and to clarify the radiomitigative potential of GT3-mobilized progenitors against supralethal doses of ionizing radiation. Our results demonstrate that the transfusion of GT3-mobilized progenitors after irradiation significantly enhanced survival of mice exposed to high radiation doses. Administration of a G-CSF antibody abrogated the efficacy of GT3 to mobilize sufficient numbers of marrow progenitors for effective, post-irradiation injury mitigation, by neutralizing GT3-induced G-CSF. Administration of GT3-mobilized peripheral blood mononuclear cells (PBMC) to irradiated mice retained GI structural integrity and reduced bacterial translocation from the gut to other organs. In brief, we report that the ability of GT3 to mobilize hematopoietic progenitors can be exploited for treatment of injuries resulting from exposure to ionizing radiation.

## Materials and Methods

### Mice

Male 6–8 week-old CD2F1 mice were purchased (Harlan Laboratories, Inc., Indianapolis, IN, USA) and housed (8 per cage) in an air-conditioned facility accredited by the Association for Assessment and Accreditation of Laboratory Animal Care-International. All mice were kept in rooms with a 12 h light/dark cycle. The mouse holding room was maintained at 21±2°C with 10–15 hourly cycles of fresh air and a relative humidity of 50±10%. Upon arrival, the mice were held in quarantine for one week. A microbiological examination of representative samples ensured the absence of *Pseudomonas aeruginosa*. Mice were provided certified rodent rations (Teklad Rodent Diet, Harlan Laboratories, Inc.) and acidified water (HCl, pH = 2.5–2.8) *ad libitum*. All animal procedures were performed according to a protocol approved by the Armed Forces Radiobiology Research Institute's (AFRRI) Institutional Animal Care and Use Committee. Research was conducted according to the Guide for the Care and Use of Laboratory Animals prepared by the Institute of Laboratory Animal Resources, National Research Council, US National Academy of Sciences [34].

### Drug preparation and administration

GT3 formulation in 5% Tween-80 in saline was purchased from Yasoo Health, Inc. (Johnson City, TN, USA). Olive oil was used as vehicle control (equivalent to the quantity of GT3) in 5% Tween-80. The final GT3 concentration was adjusted to administer a dose of 200 mg/kg in 0.1 ml. Control mice received 0.1 ml of vehicle. Mice were administered GT3 or vehicle subcutaneously (sc) at the nape of the neck with a 23 G needle attached to a Luer-Lock syringe 72 h before harvesting blood. No infections or local reactions were noted at the site of injection. AMD3100 (commercially known as plerixafor or Mozobil, Sigma-Aldrich, St. Louis, MO, USA) was diluted to 1.25 mg/ml with phosphate-buffered saline (PBS) for a dose of 5 mg/kg [35]. It has been reported that AMD3100 aids in mobilizing progenitors from bone marrow to peripheral circulation and improves the yield of progenitors when injected to mice 1 h prior to harvest [36], [37]. All donor mice, including those receiving the vehicle (olive oil), also received AMD3100 sc in a volume of 0.1 ml using a 23 G needle 1 h before blood collection to enhance mobilization [38].

The G-CSF antibody and its isotype control were purchased from R&D Systems Inc. (Minneapolis, MN, USA). Both agents were tested for 12 viral agents by BioReliance (Rockville, MD, USA) by MAP-IT (Molecular antigen PCR-identification test for mice) assay before use, and were found negative for all viral agents tested. Both reagents were diluted to a final concentration of 5,000 µg/ml with PBS. Eight h after GT3 administration, mice received a G-CSF antibody or rat IgG1 isotype control (1,000 µg/mouse in 0.2 ml, intraperitoneally, ip) with a 23 G needle.

### Irradiation

Mice were placed in ventilated Plexiglas boxes compartmentalized to accommodate eight mice per box and exposed to bilateral irradiation in the AFRRI cobalt-60 facility at a dose rate of 0.6 Gy/min. Animals were irradiated with midline doses of 11 Gy (a supralethal dose for a CD2F1 mouse with LD_50/30_ of 9.2 Gy). After irradiation, mice were returned to their cages and monitored for 30 days.

Radiation dosimetry was based primarily on the alanine/EPR (electron paramagnetic resonance) system [39], [40], currently accepted as one of the most accurate methods for relatively high radiation doses and used for intercomparison between national metrology institutions. The calibration curves (spectrometer e-Scan, Burker Biospin, Inc., Madison, WI, USA) used in dose measurements at AFRRI are based on standard alanine calibration sets purchased from the United States National Institute of Standards and Technology (NIST, Gaithersburg, MD, USA). The alanine dosimeters obtained from NIST had been calibrated in terms of absorbed dose to water using the U.S. national standard radiation sources. At AFRRI, identical alanine dosimeters were placed midline within mouse phantoms (Plexiglas 1″ diameter, 3″ length) and irradiated for predefined periods of time. Measurement of their EPR signals using the calibration curve constructed with alanine dosimeters from NIST-provided dose rates to water in the core bodies of mice. A small correction was subsequently applied for the difference in mass energy absorption coefficients between water and soft tissue.

### Blood collection and isolation of PBMC for transfusion and analysis

Donor mice were administered a single injection of GT3 (200 mg/kg, sc) and AMD3100 (5 mg/kg in 0.1 ml of PBS), 72 h and 1 h before blood collection, respectively. Donor mice were terminally anaesthetized with isoflurane (Abbott Laboratories, Chicago, IL, USA) and blood was drawn from the caudal vena cava into syringes treated with citrate dextrose (BD Diagnostics, Franklin Lakes, NJ, USA), using a 23 G needle. The ratio of the anticoagulant was 0.188 ml citrate dextrose per 1 ml of blood, according to the recommendation of the anticoagulant supplier.

To prepare PBMC for transfusion, cells were isolated by layering diluted blood (1∶1 with PBS) on histopaque-1083 (Sigma-Aldrich) and centrifuging as described earlier [33]. After the PBMC were separated from the whole blood, the cells from each treatment were pooled, washed three times with PBS with 1% fetal bovine serum (FBS) and the final cell concentration for transfusion was adjusted to 50 or 20 million cells/ml in a Hanks balanced salt solution, with 1% FBS. The PBMC were then stained with different cell-surface markers with conjugated fluorochrome to detect Sca1^+^ and c-Kit^+^ lineage negative hematopoietic cells, a portion of which are committed progenitors but retain their hematopoietic regenerative abilities. Samples were analyzed using fluorescence-activated cell sorting (FACS), as previously described [28].

### Efficacy of GT3-treated whole blood or GT3-mobilized PBMC transfusions on survival and abrogation of mitigation by G-CSF antibody administration

Donor mice were treated as discussed above and blood was harvested to isolate PBMC. Recipient mice received whole-blood transfusions or PBMC in 0.1 ml (PBS with 1% FBS), 24 h post-irradiation, via the retro-orbital sinus, using a 28 G needle. Recipient mice were monitored for survival for 30-days.

To abrogate GT3-induced progenitor mobilization, 8 h after GT3 injection, half of the donor mice received a monoclonal anti-mouse G-CSF antibody and the other half received the rat IgG1 isotype control. Blood was then harvested and PBMC were isolated as described above. Recipient mice were transfused with either whole blood or PBMC 24 h post-irradiation.

### Evaluation of intestinal bacterial translocation

To evaluate the effect of GT3-mobilized progenitor transfusion on the translocation of intestinal bacteria to heart ventricular blood, liver, and spleen, recipient mice were transfused with 5 million GT3- or vehicle-mobilized PBMC from donor mice 24 h after irradiation. Heart ventricular blood, liver and spleen tissue samples were collected aseptically on days 9, 10 and 11 post-irradiation from recipient mice and were applied immediately to 5% sheep-blood agar (SBA, supports Gram-negative and Gram-positive bacterial growth), colistin-nalidixic acid (CNA, selects Gram-positive bacteria) in 5% sheep blood, and xylose-lysine-desoxycholate agar (XLD, selects Gram-negative bacteria) media using the conventional four-dilution streaking method, with flame-sterilizing the loop between each quadrant. SBA and CNA were incubated in 5% CO_2_ at 35°C, and XLD plates were incubated at 35°C without CO_2_ for 24 h. Cultures without bacterial growth after 24 h were incubated for an additional 24 h. Plates were appropriately discarded after 48 h if no growth occurred. Where bacterial growth was observed, pertinent characteristics such as colony morphology and color were documented. Portions from representative, predominant isolated colonies from the most dilute quadrants were transferred to a fresh SBA plate and incubated in 5% CO_2_ at 35°C for 18–24 h to obtain a pure sample for species identification using a Vitek 2 Compact automated system (Biomérieux Inc., Durham, NC, USA) according to its manufacturer's validated procedure. The remaining portion of a previously selected colony was inoculated to a glass microscope slide, heat fixed, and Gram-stained for observation under oil immersion at 1000× magnification. Characteristics such as cell-wall structure, cellular shape, and cellular arrangement were recorded.

### Evaluation for bacterial endotoxin

Serum samples were obtained on days 8 and 12 post-irradiation from mice receiving either GT3-mobilized PBMC or vehicle. Samples were tested for endotoxin using the kinetic turbidimetric method with Limulus amebocyte lysate assay (LAL) (Endosafe KtA2) at Charles River Laboratories, Inc. (Charleston, SC, USA). The reported values were based on a standard curve that used endotoxin values ranging from 0.005 to 5.0 EU/ml and incorporated the reaction time of serum samples with LAL. Values that fell outside of the allowable detection limits of the assay were reported as being less than (<) or greater than (>) the standard curve values. When a sample was determined to be above the allowable detection limit of the assay, or if inhibition could not be overcome, the remaining sample was diluted further. A value less than the allowable detection limit of the assay does not necessarily equate a negative endotoxin result. Dilutions, when done correctly, should have no impact on the recovery of endotoxin.

### Endogenous colony-forming units in spleen (CFU-S)

A modified, transplant-type CFU-S_day12_ assay was performed to confirm the effect of GT3-mobilized progenitors on their spleen colony-forming capacity. GT3-mobilized PBMC infused into test mice 24 h after irradiation and the spleens were harvested on day 12 after irradiation (11 Gy). The spleens were fixed in Bouin's fixative (Polysciences, In., Warrington, PA, USA) for 2 h and then transferred to formalin [41].

### Statistical analysis

For survival data, a log-rank test was used to compare survival curves. Fisher's exact test was used to compare survival rates at the end of 30 days, with a Bonferroni correction used to account for type-I error if multiple comparisons were used. Mean values with standard errors (SE, when applicable) were reported. Analysis of variance (ANOVA) was performed to determine if there were significant differences among experimental groups. If significance was indicated, a Tukey's post-hoc test was used to determine where the significant differences occurred between paired treatment groups. All statistical tests were two-sided with a 5% significance level and performed using the statistical software SPSS version 19 (IBM, Armonk, NY, USA).

## Results

### Efficacy of GT3-mobilized whole blood in irradiated recipient mice

**Figure 1:**
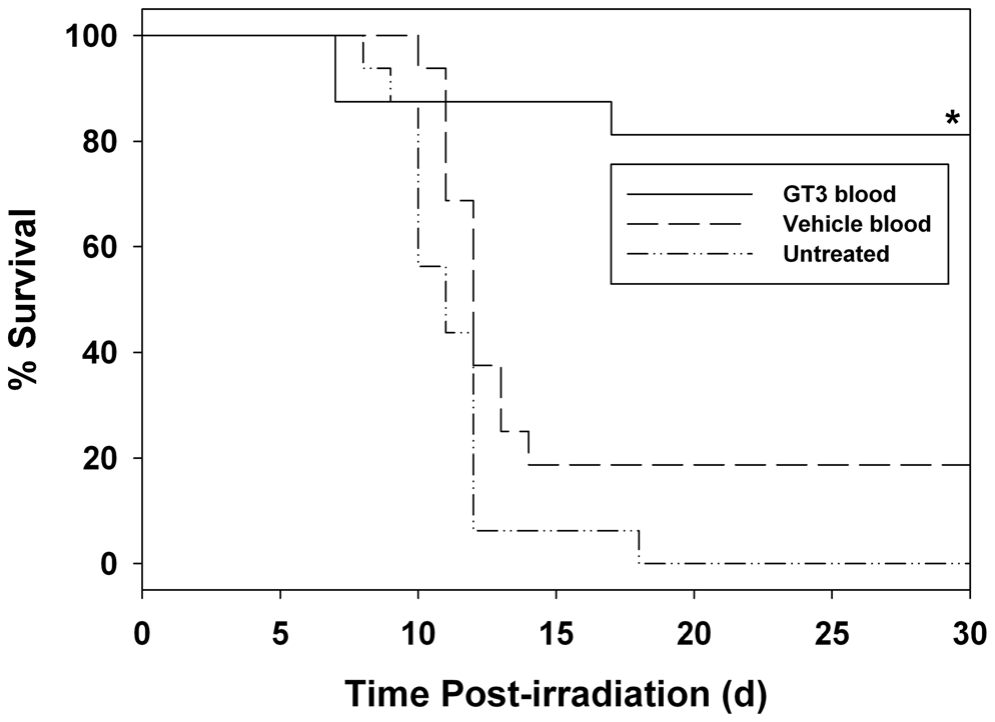
Evaluation of blood from GT3-injected mice as a radiomitigator when administered 24 h after radiation exposure (11 Gy). Mice were transfused (iv) with 100 µl of whole blood. Survival was monitored for 30 days after irradiation. *Denotes statistical significance between GT3-treated and vehicle-treated or untreated groups (*p*<0.001).

The primary objective of this study was to determine the radiomitigative efficacy of GT3-mobilized progenitors when transfused into mice 24 h following a potentially lethal 11 Gy ^6^°Co γ-radiation exposure. Recipient mice received 100 µl of blood from GT3- or vehicle-treated mice via the retro-orbital sinus 24 h post-irradiation. All experiments had a third group of irradiated mice that received neither blood nor PBMC; these irradiated, but untreated mice served as radiation controls. All treatment groups consisted of 16 mice and experiments were repeated at least twice. Data presented in Figure 1 demonstrate that administration of the whole blood from GT3-treated donors had 81% survivors compared to 19% survivors for the mice receiving vehicle-treated whole blood. All mice in the untreated group died by 17 days post-irradiation. Significantly enhanced survival benefit was observed in the mice treated with whole blood from GT3-treated donors when compared to both mice transfused with vehicle-treated donor whole-blood and untreated mice (*p*<0.001).

### Efficacy of GT3-mobilized PBMC in irradiated recipient mice

**Figure 2:**
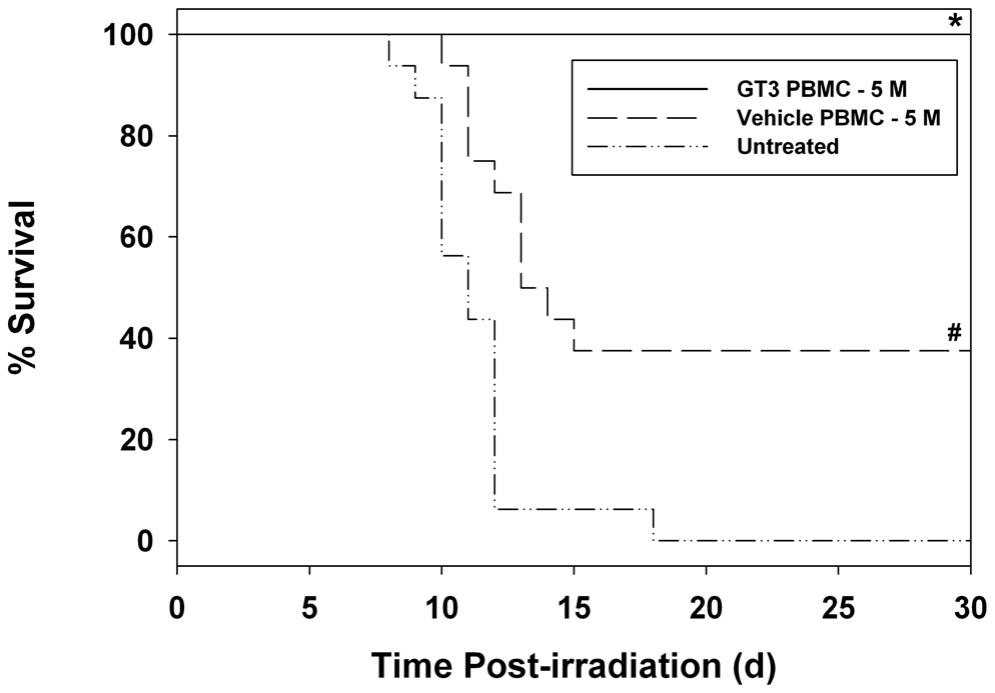
Evaluation of GT3-mobilized PBMC as a radiomitigator when administered 24 h after irradiation (11 Gy). Mice were transfused (iv) with 5 million PBMC in a volume of 100 µl of PBS containing 1% FBS. Survival was monitored for 30 days after irradiation. *Denotes statistical significance between GT3-mobilized PBMC-treated and vehicle-mobilized PBMC-treated or untreated groups (*p*≤0.001). #Denotes statistical difference between vehicle-mobilized cell-treated and untreated groups (*p* = 0.007).

We hypothesized that GT3, which stimulates high levels of G-CSF, will mobilize hematopoietic progenitors to the peripheral circulation, and that this progenitor-enriched blood, when collected and infused into lethally irradiated mice, will provide a survival benefit. Approximately 5 million PBMC were obtained from a sample of 600 µl whole blood. Each million PBMC collected from GT3-injected mice contained approximately 1,800 Lin^−^ Sca-1^+^ and 2,600 Lin^−^ c-Kit^+^ cells. For the vehicle group, these values were approximately 1,100 and 1,250, respectively. Data presented in Figure 2 demonstrate that mice receiving 5 million GT3-mobilized PBMC provided 100% survival, a significant improvement over a 38% survival of the mice that received vehicle-mobilized PBMC (*p*<0.001). All untreated mice died day 17. Most of these deaths occurred between days 7 and 17 post-irradiation. Interestingly, mice transfused with 5 million PBMC from the vehicle-treated donors also had significantly increased survival over untreated mice (*p*<0.007), suggesting that PBMC from vehicle-injected mice had some radiomitigative potential. This was not surprising since such samples contained progenitors as demonstrated by flow cytometric immunophenotyping (data not presented).

### Effect of G-CSF antibody administration to donor mice on efficacy of GT3-mobilized whole blood or PBMC in irradiated recipient mice

**Figure 3:**
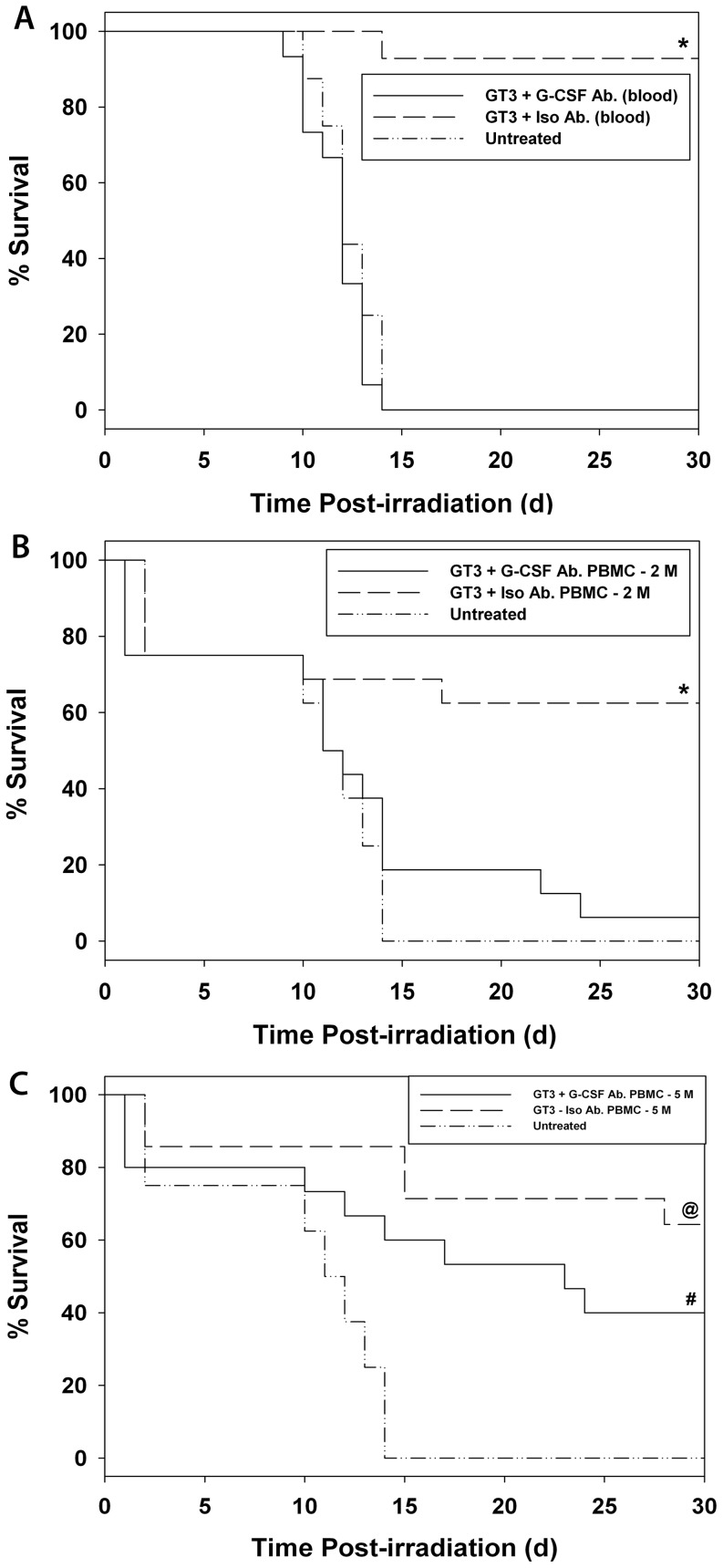
Evaluation of blood (A) or PBMC (B and C) from donor mice injected with GT3 and G-CSF antibody or isotype in irradiated mice. Donor mice were injected (sc) with 200 mg/kg of GT3, then G-CSF antibody or isotype (1,000 µg/mouse, ip) 8 h after GT3 injection. Blood was collected 64 h after G-CSF antibody/isotype injection. Recipient mice were injected 24 h after 11 Gy radiation exposure with A) whole blood, or B) 2 million cells or C) 5 million cells in 100 µl of PBS containing 1% FBS. *Denotes significant differences between recipients receiving cells from animals treated with isotype versus G-CSF antibody or untreated control groups (p<0.01). @ Denotes significant difference between isotype-treated and untreated groups (p<0.001). # Denotes significant difference between G-CSF antibody-treated and untreated groups (p = 0.014).

We were interested to know the efficacy of PBMC recovered from GT3-injected mice receiving an exogenous G-CSF antibody because the mobilization of progenitors is a GT3-induced, G-CSF-based phenomenon. Mice were injected with GT3 and, 8 h after injection, mice were administered either a G-CSF antibody or its isotype. Blood samples were collected after 72 h of GT3 injection for PBMC separation. As mentioned earlier, AMD3100 was administered to all donor groups 1 h prior to blood collection in order to enhance mobilization. As shown in figure 3A, mice treated with whole blood from the GT3- and isotype-injected donors had 93% survivors compared to the 0% survivors in the group of mice, which received blood from GT3- and G-CSF antibody-injected mice (*p*<0.01). Mice that received transfusions from the G-CSF antibody-administered donors had a survival curve that closely matched that of the untreated group; both groups had 100% mortality by day 14. Data presented in figure 3B show that mice injected with 2 million GT3-mobilized PBMC with isotype control had a significantly higher survival rate (64%) compared to both the untreated mice (0%) and mice administered GT3-mobilized PBMC obtained from GT3- and G-CSF antibody-injected mice (6%) (*p*≤0.001). All untreated mice died by day 13 after irradiation. This experiment along with data from the above experiment with blood demonstrated the neutralizing efficacy of a G-CSF antibody on G-CSF-mediated mobilization of progenitors by GT3.

We repeated the above experiment using 5 million PBMC. Mice transfused with GT3-mobilized PBMC with isotype control had a significantly higher rate of survivors (64%) compared to untreated mice (0% survivors, *p*<0.001) (figure 3C). Mice transfused with PBMC obtained from GT3- and G-CSF antibody-injected mice had 40% survivors significantly higher than untreated mice (*p*<0.014). There was no significant difference in survival between mice receiving PBMC, which were obtained from G-CSF antibody- or isotype-injected mice (in addition to GT3). This result further supports data presented in Figure 2 that 5 million PBMC from mice (irrespective of treatment) has radiomitigative potential, though to a lesser extent than cells obtained from GT3-treated animals.

### Effect of GT3-mobilized progenitor cell transfusion on the translocation of intestinal bacteria in irradiated mice

**Figure 4:**
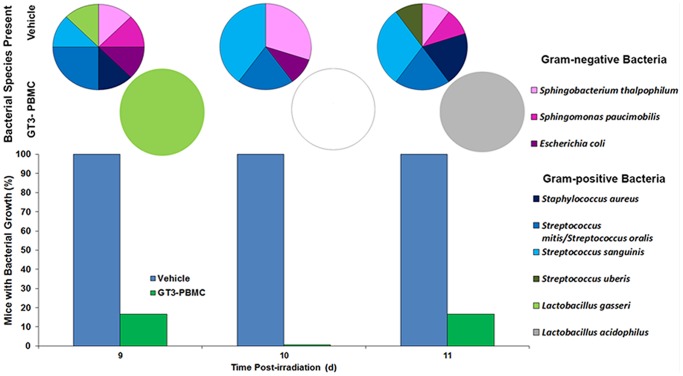
Bacterial translocation in GT3-mobilized PBMC recipient mice. Mice were transfused with 5 million PBMC from GT3-injected mice and compared with control group. Heart blood, spleen, and liver samples were collected aseptically on days 9, 10, and 11 days post 11 Gy ^6^°Co γ-irradiation. Samples were inoculated onto agar media using the conventional four-dilution-streak method and monitored for bacterial growth. Isolated bacteria were identified with Vitek 2 Compact automated system.

To analyze the potential anti-infective effect of transfused GT3 mobilized PBMC relative to bacterial translocation across the gut bacteria to other organs, we transfused supralethally irradiated mice with 5 million GT3-mobilized PBMC and compared patterns of subsequent bacterial infections with control groups that had received no treatment. Heart blood, liver, and spleen samples were aseptically collected on days 9, 10 and 11 post-irradiation, inoculated to agar media, incubated for 24 or 48 h. Isolated bacteria were identified. All control irradiated mice receiving no cell administration sampled at three time points (18 out of 18; 100%) had one or more bacterial species infecting the tissue samples (Table 1). This number was higher compared with bacterial isolation from only 2 out of 18 mice (one on day 9 and another on day 11), which were treated with GT3-mobilized PBMC. The two mice treated with GT3-mobilized progenitors had isolates of a single species, whereas 11 control mice had a single isolated species and seven control mice had two isolated species, i.e., a polymicrobial infection. Data presented in Table 2 demonstrate that relative bacterial concentrations in control-group animal organs were higher (+++ and ++++) than in GT3-mobilized PBMC-treated mice (+). The two isolates from GT3-mobilized PBMC-injected mice were Gram-positive species (*Lactobacillus gasseri* and *Lactobacillus acidophilus*). The vehicle-treated group had three Gram-negative species (*Sphingobacterium thalpophilum*, *Sphingomonas paucimobilis*, and *Escherichia coli*) and five Gram-positive species (*Staphylococcus aureus*, *Streptococcus mitis/Streptococcus oralis*, *Streptococcus sanguinis*, *Streptococcus uberis, and L. gasseri*) (Figure 4).

**Table 1:** Details of bacterial translocation in mice given 11 Gy ^6^°Co-γ radiation and administered 5 million GT3-mobilized progenitor cells.

Details of mice analyzed	Number of GT3-PBMC-Treated Mice	Number of Vehicle-PBMC-Treated Mice
Mice cultured	18	18
Mice septic	2	18
Single isolated bacterial species	2	11
Two isolated bacterial species	0	7
One Gram-negative + one Gram-positive species	0	5
Two Gram-positive species	0	2
Two Gram-negative species	0	0

**Table 2:** Details of bacterial growth in samples of heart blood, liver and spleen collected from mice on 9, 10, and 11 d post-irradiation.

Group	Time post-irradiation (day)	Mouse ID number^a^	Number of species identified	Bacterial Growth in Heart Blood^b^	Bacterial Growth in Liver^b^	Bacterial Growth in Spleen^b^
Control	9	1	1	+	NG	NG
Control	9	2	1	++	++	+++
Control	9	3	2	++	+	+
Control	9	4	1	NG	+	++
Control	9	5	1	+	+++	+
Control	9	6	1	NG	++	NG
Control	10	13	2	+	++++	+++
Control	10	14	1	++	+	NG
Control	10	15	2	+	+++	+++
Control	10	16	1	+	+	+
Control	10	17	2	+	++	++
Control	10	18	1	+++	++++	++
Control	11	25	2	+	+	+
Control	11	26	2	+++	+	+
Control	11	27	1	+	++	+
Control	11	28	1	+	+	+
Control	11	29	2	++	++	+
Control	11	30	1	+	+	+
GT3-PMBC^c^	9	8	1	+	NG	NG
GT3-PMBC^c^	11	33	1	NG	NG	+

a At each time point, *n* = 6 mice sampled per treatment group.

b + indicates the relative concentration of bacterial colonies in the first quadrant only of the agar isolation medium, using the four-dilution streak method, ++++ indicates bacterial growth in the fourth quadrant of the plate, farthest from the primary inoculation, indicating the highest concentration of bacteria. NG =  no growth. The GT3-PBMC mice, in which no bacteria were detected in tissues, were not included in the table.

c GT3-PBMC mice received transfusion of five million PBMCs (iv) 24 h post-irradiation (11 Gy cobalt-60 gamma-radiation at 0.6 Gy/min dose rate).

### Effect of GT3-mobilized progenitor cell transfusion on levels of bacterial endotoxin

Due to limited sample volume, serum had to be diluted 1∶100 for the assay. The first time this experiment was performed, a dilution factor of 1∶100 was used, resulting in concentrations outside the standard curve and detection limit (<0.5 EU/ml) for all 12 samples (6 each from vehicle control and GT3-mobilized PBMC recipient groups). This experiment was repeated using the same treatment groups but utilizing smaller dilution factors for the serum samples to obtain endotoxin concentrations that fell within the detection limit. Endotoxin positive control samples also were incorporated into the samples analyzed (Lonza Group Ltd, Walkersville, MD USA). Serum samples were collected from 2 moribund mice on day 8. The serum samples from the remainder of the vehicle-treated mice (*n* = 6) and the mice transfused with GT3-mobilized PBMC (*n* = 7) were collected 12 days post-irradiation. Out of six vehicle control mice, one had 1,672 EU/ml endotoxin, three had <2.5 EU/ml and the remaining two had <0.5 EU/ml. Of six GT3-mobilized PBMC recipient mice, one had 3.11 EU/ml and the remaining five had <0.5 EU/ml.

### GT3-mobilized progenitors enhance hematopoietic recovery

**Figure 5:**
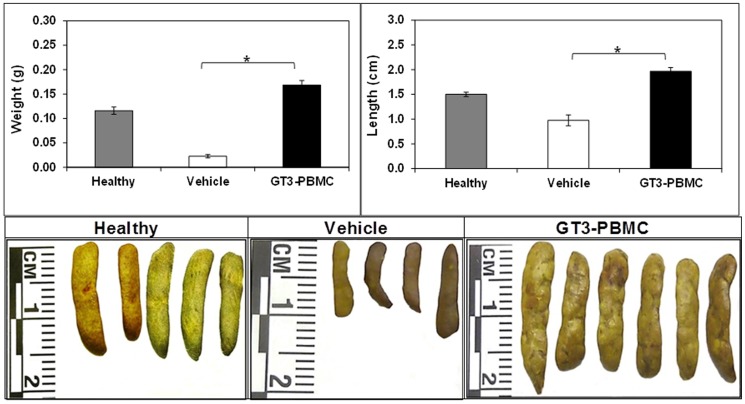
Effects of TS administration on colony forming unit-spleen in CD2F1 mice. GT3 mobilized PBMC were administered to CD2F1 mice iv 24 h after irradiation. Spleens were harvested 12 days after irradiation (11 Gy at 0.6 Gy/min).

To confirm the radiomitigative efficacy of GT3-mobilized progenitor administration on hematopoiesis in mice given 11 Gy of ionizing radiation, a conventional CFU-S macroscopic assay was performed. The number of CFU-S is considered to be an important indicator of a functional, trilineal hematopoietic system and in turn, vital in terms of survival. Irradiated mice, which were administered GT3-mobilized progenitors, had higher numbers of CFU-S that coalesced with each other, making precise counting difficult, compared to mice receiving cells from vehicle-treated donors. These control mice had 2 – 8 CFU-S per spleen. These spleens were larger in size and weight compared to vehicle-injected mice on day 12 post-irradiation, indicating hematopoietic restoration ability of the splenic progenitor cells (Figure 5).

## Discussion

For patients who do not respond well to cytokine therapies, an allogeneic stem-cell transplantation is a viable but clearly difficult therapeutic option. In mass-casualty situations, finding multiple human leukocyte antigen (HLA)-matched donors for transplantations would be undoubtedly extremely difficult. Currently, recombinant G-CSF is used clinically to mobilize CD34^+^ cells into the blood of donors in order to collect progenitor-enriched cell fractions for subsequent transfusions in the treatment of severely immunocompromised patients [42].

We are developing a new strategy to treat individuals who are at high risk for exposure to acute, high doses of ionizing radiation (e.g., military personnel, first responders). We suggest that GT3 will mobilize high-quality hematopoietic progenitors following its administration. Our strategy involves the mobilization of progenitors by GT3 and the subsequent collection of whole blood or progenitor-enriched blood cell fractions well before an ionizing radiation exposure occurs. We have tested the efficacy of blood or PBMC transfusion against a supralethal dose of radiation (11 Gy) in CD2F1 mice. This relatively high radiation dose causes hematopoietic as well as GI injury [43]. GT3-mobilized PBMC (also whole blood) mitigated radiation injury in mice against 11 Gy of ^6^°Co γ-radiation. Such progenitor mobilization has been reported for tocopherol succinate (α-tocopherol ester) [44]. Unlike tocopherol succinate, GT3 is soluble in a FDA-approved excipient (5% Tween-80 in saline) making it more user-friendly for possible clinical use. In rodents, mobilization of progenitors by tocols is as efficient as G-CSF [33]. However, we have not tested pharmaceutical grade GT3 for G-CSF induction and mobilization of progenitors and therefore cannot attest to their comparable characteristics.

The next set of experiments was performed to determine the role of G-CSF antibody administration on mobilization of progenitors by GT3 in donor mice. Mice were administered either whole blood or PBMC from donors that received GT3 administration followed by either a G-CSF antibody or an isotype control prior to blood harvest. As shown in figures 3A and 3B, the mice that received whole blood or PBMC collected from isotype-injected mice had significant survival benefits compared to mice receiving blood or PBMC from G-CSF antibody-injected animals, suggesting that G-CSF antibody neutralized G-CSF that was induced by GT3, and thereby inhibited progenitor mobilization. When we administered 5 million PBMC from donor isotype-injected mice to irradiated recipient mice, the transfused PBMC improved the survival of those recipients; whereas PBMC obtained from G-CSF antibody-injected animals also benefitted recipients though to a lower degree. This observation suggests that 5 million PBMC are capable of mitigating radiation injury to some extent. As stated above under the Results section, in another experiment, 5 million PBMC from control animals aided in recovery of irradiated recipients, suggesting that such PBMC have some mitigative efficacy when injected into irradiated mice.

Lethal doses of radiation are known to cause significant gastrointestinal injury that promotes bacterial translocation from the gut into the lymphatics and blood and into different organs [45], [46]. This bacterial translocation is considered to be an extremely important pathophysiological process associated with potentially fatal radiation-induced injury [47], [48]. Consequently, we also investigated the effects of GT3-mobilized progenitors on this radiation-induced pathology of the gut. Our results suggest that the GT3-mobilized progenitor-transfused mice were less likely to translocate sepsis-caused bacteria across the wall of the gut than were control mice infused with vehicle-mobilized progenitors; i.e., not only does transfusion of GT3-mobilized progenitors inhibit translocation but this treatment also reduces polymicrobial infections, particularly by Gram-negative species. These findings are consistent with older reports demonstrating the effect of α-tocopherol in attenuating the incidence of bacterial translocation in rats [49]. We recently have reported that tocopherol succinate, tocopherol-succinate-mobilized progenitors, and myeloid progenitor treatment inhibit bacterial translocation in ^6^°Co γ-irradiated CD2F1 mice [50]–[52]. These results support our current findings regarding GT3-mobilized progenitors. Additionally, we have observed high levels of endotoxin in vehicle-mobilized progenitor recipients. It is important to note that endotoxin is associated with mice infected with Gram-negative bacteria only. Thus, the numbers of mice with high levels of endotoxin do not reflect the total number of mice, which develop bacterial infection.

Jejunum histopathology demonstrated that transfusion of GT3-mobilized progenitors into irradiated mice mitigates radiation-induced GI injury. There was significant recovery in GT3-mobilized progenitor recipients compared to untreated control and PKH26-labelled mobilized cells were detected by fluorescence microscopy in various organs of recipient mice (data not presented). Similar recovery of radiation-induced GI injury was observed earlier with tocopherol succinate [50].

There are a number of major advantages observed in the mouse model described here that make GT3-mobilized progenitors attractive for the treatment of patients/casualties with ARS: a) GT3-mobilized progenitor therapy is essentially non-toxic, b) GT3-mobilized progenitor therapy clearly allows for a broader treatment range (in terms of the extent of radiation exposure) for treating both the hematopoietic and gastrointestinal-related subsyndromes of ARS, c) GT3 is stable at room temperature and suitable for long-term storage, d) GT3 may replace currently used G-CSF for progenitor mobilization in the clinic, and e) GT3 can be administered via a FDA-approved vehicle [27], [29], [43], [53]–[55]. In comparison, G-CSF is requires continuous cold storage, making its availability difficult during any disaster scenario. These characteristics make GT3-mobilized progenitors a suitable candidate as a bridging therapy for acute radiation victims that can be administered in the field with minimal infrastructure requirements. With further preclinical development in large animals and clinical trials in future, we may be able to provide an appropriate protocol for the clinical management of individuals suffering from high doses of ionizing radiation.

In summary, GT3 has been shown to be attractive and promising radiation countermeasure using mouse [14] and nonhuman primates (unpublished observation) models. It induces high levels of G-CSF in circulation within 24 h of sc administration that leads to mobilization of marrow progenitors into peripheral blood. This study suggests that mobilized progenitors mitigate radiation injury in irradiated mice. Efficacy of such cells can be abrogated by administering a G-CSF antibody to donors, suggesting that mobilization of progenitors by GT3 is a G-CSF-dependent phenomenon. Administration of PBMC from GT3-injected mice inhibits bacterial translocation from intestine to blood and other organs, suggesting that, in addition to hematopoietic recovery, such cells also provide GI recovery in irradiated recipients. To the best of our knowledge, this is the first report demonstrating that G-CSF-mediated mobilization of progenitors by GT3 is effective as a radiation mitigator when used to transplant critically irradiated and injured animals, and that the efficacy of such cells can be abrogated by administering a G-CSF antibody. This treatment option appears attractive based on studies in mouse model.
